# Combined ultra-low input mRNA and whole-genome sequencing of human embryonic stem cells

**DOI:** 10.1186/s12864-015-2025-z

**Published:** 2015-11-12

**Authors:** Florian Mertes, Björn Lichtner, Heiner Kuhl, Mirjam Blattner, Jörg Otte, Wasco Wruck, Bernd Timmermann, Hans Lehrach, James Adjaye

**Affiliations:** Department of Vertebrate Genomics, Max Planck Institute for Molecular Genetics, Ihnestr. 63-73, 14195 Berlin, Germany; Next Generation Sequencing Group, Max Planck Institute for Molecular Genetics, Ihnestr. 63-73, 14195 Berlin, Germany; Institute for stem cell research and regenerative medicine, Medical Faculty, Heinrich Heine University, Moorenstr. 5, 40225 Düsseldorf, Germany; Molecular Exposomics, Helmholtz Zentrum München, Ingolstädter Landstr. 1, 85764 Neuherberg, Germany

**Keywords:** Next generation sequencing, RNA-seq, Whole-genome sequencing, Ultra-low input sequencing, Single cell, Pluripotency, Embryonic stem cells

## Abstract

**Background:**

Next Generation Sequencing has proven to be an exceptionally powerful tool in the field of genomics and transcriptomics. With recent development it is nowadays possible to analyze ultra-low input sample material down to single cells. Nevertheless, investigating such sample material often limits the analysis to either the genome or transcriptome. We describe here a combined analysis of both types of nucleic acids from the same sample material.

**Methods:**

The method described enables the combined preparation of amplified cDNA as well as amplified whole-genome DNA from an ultra-low input sample material derived from a sub-colony of in-vitro cultivated human embryonic stem cells. cDNA is prepared by the application of oligo-dT coupled magnetic beads for mRNA capture, first strand synthesis and 3’-tailing followed by PCR. Whole-genome amplified DNA is prepared by Phi29 mediated amplification. Illumina sequencing is applied to short fragment libraries prepared from the amplified samples.

**Results:**

We developed a protocol which enables the combined analysis of the genome as well as the transcriptome by Next Generation Sequencing from ultra-low input samples. The protocol was evaluated by sequencing sub-colony structures from human embryonic stem cells containing 150 to 200 cells. The method can be adapted to any available sequencing system.

**Conclusions:**

To our knowledge, this is the first report where sub-colonies of human embryonic stem cells have been analyzed both at the genomic as well as transcriptome level. The method of this proof of concept study may find useful practical applications for cases where only a limited number of cells are available, e.g. for tissues samples from biopsies, tumor spheres, circulating tumor cells and cells from early embryonic development. The results we present demonstrate that a combined analysis of genomic DNA and messenger RNA from ultra-low input samples is feasible and can readily be applied to other cellular systems with limited material available.

## Background

Within recent years an overwhelming number of specific methods and protocols emerged for next-generation sequencing [1]. Amongst them, transcriptome as well as whole-genome sequencing were of prime interest. Both sequencing methods have tremendously accelerated our understanding of both the more dynamic function of RNAs and the more static composition of the genome within a functional cell. Transcriptome sequencing focuses on deciphering the complex expression pattern of RNAs [2, 3], therefore identifying novel expressed RNAs and transcript variants as well as isoforms which in turn lead to a better understanding of cell regulation, function and networks. Whole-genome sequencing has for example highlighted insights into the subtle differences amongst the human population [4, 5] or major genomic re-arrangements found in cancer cells [6] with both having a significant impact on cell fate and the living organism.

Major improvements for the preparation of sequencing libraries for RNA-seq as well as DNA-seq have emerged [7, 8]; continually reducing the input amount needed which is generally in the microgram range. Within recent years the field of single-cell sequencing for transcriptome and genome sequencing has advanced significantly [9, 10]. There are already many examples available were either RNA [11–15], or DNA [8, 16, 17] have been analyzed down to the single-cell resolution. Recently, studies with the combined analysis of the genome and transcriptome of the same cell have also been published [18, 19]. This is especially advantageous for applications where only a small fraction of the sample should be analyzed, or more importantly, where the sample is composed of a few cells only. Such scenarios include for example early embryonic development which starts from a single cell expanding to a few dozen cells within the first developmental stages [20]. The elucidation of intra-tumor heterogeneity in biopsies [21, 22] as well as in *in-vitro* grown primary tumor spheres [23], or the characterization of circulating tumor cells [24] rely on the analyses of limited cell material. In addition, *in-vitro* cultured stem cells from both mouse and human are limited in the number of cells if sub-population and sub-colony differences in terms of gene expression are under investigation. For all settings, already subtle changes in genome integrity can have a major impact on the expression and regulation of RNAs, and proteins within cells.

Despite the advancements for both areas of sequencing minute amounts of either RNA or DNA, an assay enabling the combined sequencing of RNA and DNA from the very same sample still in the ultra-low input range would add to our understanding of the regulation and developmental processes affected by both, the function of genome integrity as well as RNA expression and gene function.

Here we describe a method which enables the preparation of whole transcriptome amplified cDNA as well as the generation of whole-genome amplified DNA from the same ultra-low input material derived from a sub-colony of *in-vitro* cultivated human embryonic stem cells. Firstly, whole transcriptome amplified cDNA was prepared from mRNA only by using oligo-dT coupled magnetic beads, following cDNA synthesis, 3'-tailing and PCR amplification. Secondly, after magnetic coupling of the mRNA/oligo-dT beads, whole-genome amplified DNA was prepared from the retained DNA by Phi29 mediated amplification. Both, the amplified cDNA as well as DNA were subjected to standard procedures for multiplex short fragment library preparation enabling Illumina sequencing. Using this approach, both the transcriptome as well as the genome of the same sample could be analyzed on both levels of nucleic acids present in cells, the RNA and DNA.

## Results

### Ultra-low input RNA sequencing

**Fig. 1:**
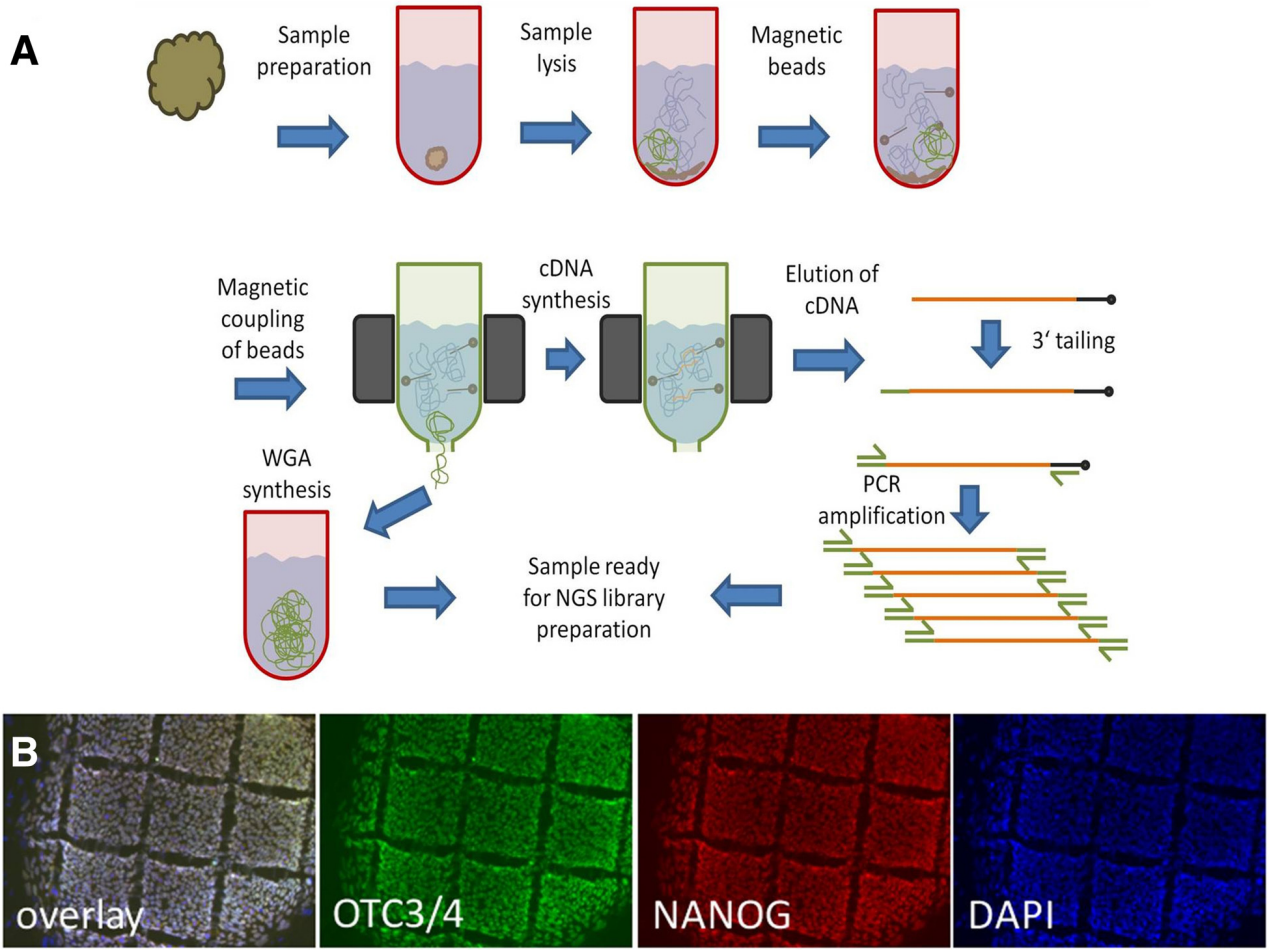
Method overview for combined sequencing of mRNA and whole genome DNA. **a** Schematics for sequencing of ultra-low input DNA and mRNA from a single cell-colony sample. **b** OCT3/4, NANOG and DAPI staining of cultured human embryonic stem cells. Rectangular cuts indicating size of sample applied (150–200 cells each). Scale bar =200 μm

In brief, cells for RNA-seq were collected from human embryonic stem cells (hESCs) serving as biological samples. Colonies of hESCs were mechanically dissociated into 200 μm × 200 μm square fragments consisting of 150–200 cells (Fig. 1b). The undifferentiated and pluripotent state of the cells was verified by microscopic assessment of morphology (small, densely-packed cells with high nuclei:cytoplasm-ratio growing in a homogeneous monolayer) and positive immunocytochemical co-staining for the well-established transcription factors and hESC-markers OCT3/4 and NANOG [25] (Fig. 1b).

The picked sub-colony fragment was directly transferred into lysis buffer. After cell lysis, the solution was supplemented with oligo-dT coupled magnetic micro-beads and transferred to columns placed in a magnetic field for further processing. To selectively enrich mRNA out of the total RNA, cDNA synthesis was performed with oligo-dT coupled magnetic beads. After on column cDNA synthesis, beads with cDNA were retained by centrifugation followed by 5′-tailing and PCR amplification. The size distribution of amplified cDNA ranged from 200–3000 base pairs. PCR products were fragmented by sonication to 150 to 300 base pairs and multiplex fragment library preparation was performed for paired-end Illumina sequencing. Figure 1 gives an overview of the developed methodology.

In this study we report data from sequencing of two hESC samples in the low sub-colony range (150–200 cells, Fig. 1b) which were analyzed by 100 base pair paired-end sequencing on a single flow cell on an Illumina HiSeq instrument. We obtained 314.2 million raw reads on a single lane, after barcode mapping for sample allocation we obtained 65.2 million reads for RNA-seq sample 1 and 58.8 million reads for RNA-seq sample 2 respectively. Furthermore 190.5 million reads belonged to whole genome sequencing performed for sample 1. The RNA-seq reads were mapped with Tophat resulting in 58.9 and 54.7 million mapped reads (90.2 and 93.5 %) correspondingly. The number of duplicate reads was found to be 1.9 % for the WGA-DNA sample and 54.6 and 52.8 % for the RNA-seq sample 1 and 2 respectively. Duplicate read counts were based on mapped reads with the same start and end point. In total 11,755 Refseq genes with a read coverage of ≥ 5 reads were identified (sample 1: 8523; sample 2: 10,908; overlap: 7676) in both hESC samples.

**Fig. 2:**
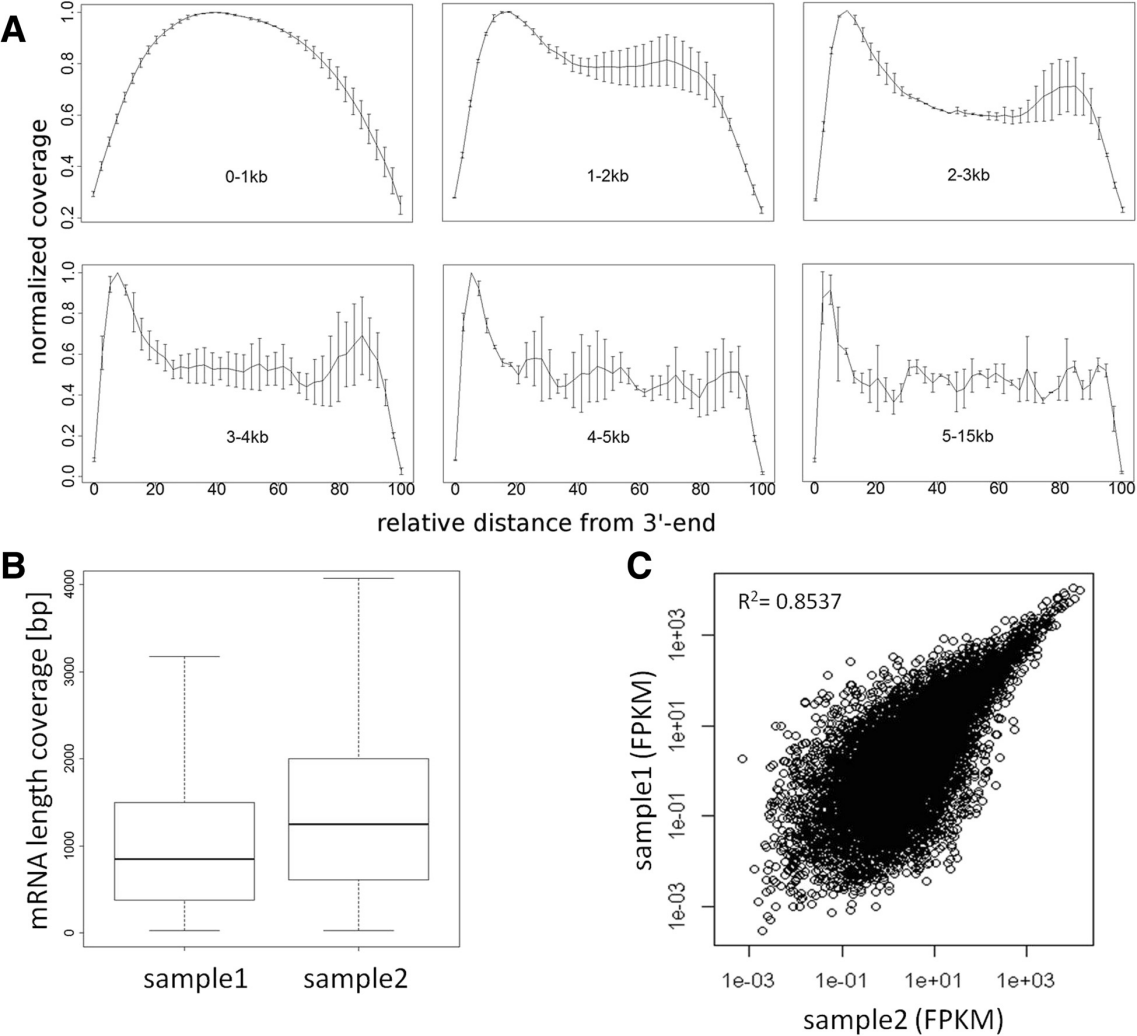
Comparison of RNAseq data in terms of transcript identification. **a** Read coverage across transcripts with coverage for transcripts of sizes 0 kb-1 kb, 1 kb-2 kb, 2 kb-3 kb, 3 kb-4 kb, 4 kb-5 kb and 5 kb-15 kb. Transcripts were divided into 40 equally sized bins and coverage was averaged over all transcripts of the dedicated size interval. Mean values of mRNA sample1 and sample2 are displayed, error bars denoting standard deviation of samples. **b** Boxplot of total length of sequenced Refseq genes for RNAseq sample 1 and sample 2. **c** Correlation plot for RNAseq sample 1 and sample 2 in terms of FPKM values for expressed Refseq genes

Next imperative parameters for RNA-seq were determined based on the Refseq dataset, specifically the total length of mRNA showing sequence coverage and coverage distribution along the 5'- to 3'-orientation. Since we performed cDNA synthesis by oligo-dT priming it is common sense to observe a bias towards the 3′-end of genes, especially for long transcripts (Fig. 2a). The median length of Refseq cDNAs observed were around 900–1100 base pairs ranging from approximately 450 to 2000 base pairs (lower and upper quartile) with single cDNAs longer than 10 kb (Fig. 2b). The average coverage distribution along the 5'- to 3'-orientation of Refseq genes was calculated for transcript size intervals of 0–1 kb, 1–2 kb, 2–3 kb, 3–4 kb, 4–5 kb and 5–15 kb. For transcripts in the range of 1–2 kb normalized coverage was almost 80 % over the full transcript length with decreasing coverage towards the last 15 % of bases at the 5′-end. For transcripts ranging from 2–5 kb normalized coverage was at least 50 % (Fig. 2a). Subsequently we evaluated the correlation between both RNA-seq samples. This was done by comparing FPKM values obtained for expressed Refseq genes resulting in a Pearson’s correlation factor of 0.85 for RNA-seq sample 1 and sample 2 (Fig. 2c).

**Fig. 3:**
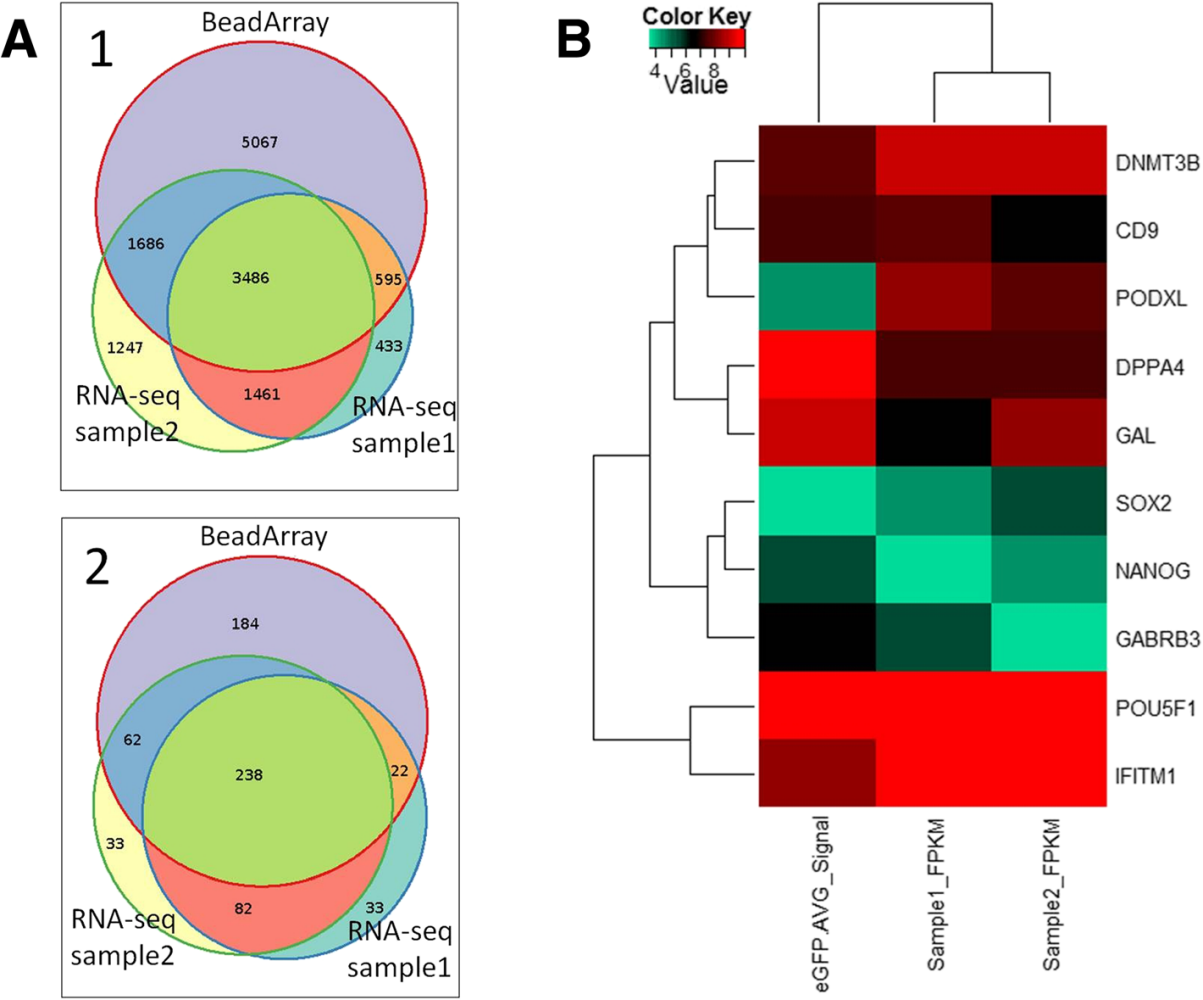
Overlap between microarray and sequencing measurements. **a.1** Venn diagram displaying overlap of significant genes found in the Illumina microarray and next-generation sequencing (NGS) experiments. Genes expressed with Illumina detection *p*-value < 0.05 in the microarray experiment or FPKM > 0.5 in the NGS experiments were considered significant. Genes were compared via HGNC gene symbol annotation. **a.2** Venn diagram displaying overlap of significant CPDB categories found in the Illumina microarray and deep sequencing experiments. Significant genes from microarray and NGS experiments were analysed with the CPDB functional annotation tool and categories with a *q*-value <0.05 were considered significant. **b** Pluripotency associated genes from microarray experiment and NGS experiments of sample1 and sample2 were compared. Logarithmic values (base 2) of Illumina average signals and FPKM values from expressed genes (detection *p*-value < 0.05, FPKM > 0.5) were quantile normalized and subjected to cluster analysis via R heatmap2 function using Euclidean distance as distance measure

To further evaluate the RNA-seq data we used expression analysis performed with an Illumina BeadArray with the same hESC line. The BeadArray experiments were performed with the appropriate amount of mRNA in comparison to the low input RNA-seq experiments. A comparison of expressed Refseq genes for both RNA-seq samples and the Illumina BeadArray showed a high degree of concordance for both methods. For analysis only genes were included which gave rise to FPKM >0.5 for NGS data and *p*-value <0.05 in BeadArray and were considered significant. In total 13,630 genes were identified in both RNA-seq samples whereas the BeadArray identified 10,834 genes. The total overlap between both sequencing experiments and the BeadArray was found to be 3486 Refseq genes. Moreover the overlap for RNA-seq sample 1 and BeadArray was found to be 4081 and for RNA-seq sample 2 to be 5172, respectively (Fig. 3a). Next, a Consensus Pathway Data Base (CPDB) overrepresentation analysis was performed to identify congruence of BeadArray and NGS experiments in terms of overlapping genes and categories (Fig. 3a). Significant genes from all experiments were analyzed in CPDB for categories using pathways from KEGG, Reactome, BioCarta and Wikipathways and compared for categories with a *p*-value threshold of 0.05. In total 506 categories were identified for the BeadArray experiment and 375 and 415 categories for RNA sequencing sample 1 and sample 2 respectively. Overall overlap for all experiments was found to be 238 and for BeadArray and combined RNA samples 322 categories. For RNA sequencing sample 1 and RNA sample 2 only the overlap was found to be 320 categories.

**Fig. 4:**
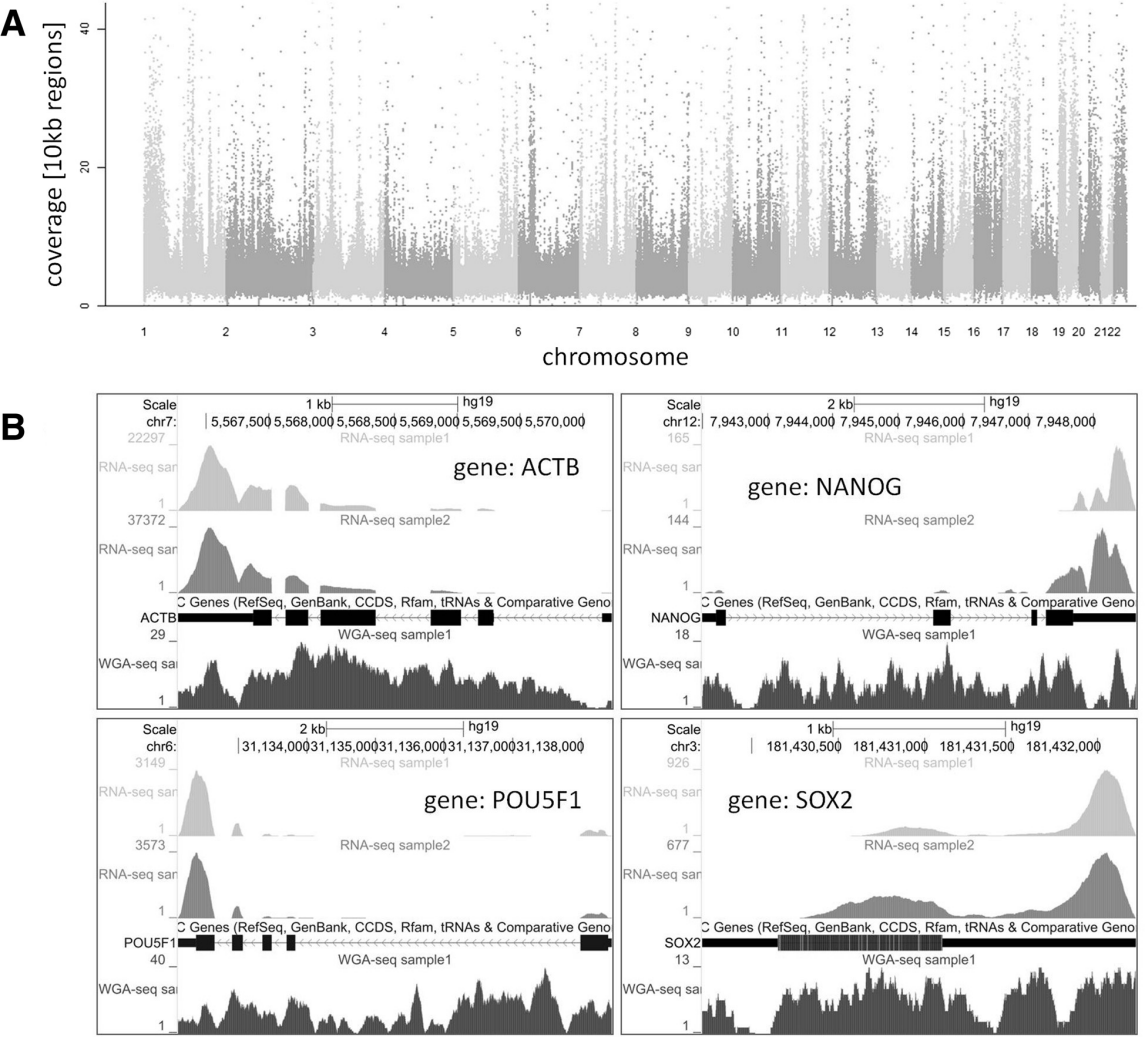
Read coverage of genome sequencing and RNA-seq data. **a** Read coverage of genomic DNA is visualized in a Manhattan plot showing coverage of 10 kb regions over chromosomes. **b** Example of mapped reads for selected stem cell marker genes (*NANOG*, *POU5F1, SOX2*,) and housekeeping gene (*ACTB*) for both RNA-seq samples (light grey: RNA-seq sample1 and medium grey: RNA-seq sample2) and WGA DNA sample (dark grey). The aligned RNA-seq reads resemble the exon structure depicted below

To evaluate pluripotency of hESC samples in BeadArray and RNA sequencing experiments common pluripotency marker genes were compared. Comparison was performed after normalization of gene expression by graphical representation analysis (Fig. 3b). The differential analysis of gene expression showed highest similarity between both RNA samples followed by BeadArray. Analysis of expressed genes among all samples showed clustering of genes in groups of two for *DNMT3B* and *CD9*, *SOX2* and *NANOG* and *POU5F1* (*OCT3/4*) and *IFITM1* with very similar gene expression in BeadArray and both RNA sequencing samples. Examples of sequencing coverage for single pluripotency marker genes (*NANOG, POU5F1* and *SOX2)* as well as housekeeping gene (*ACTB*) are shown in Fig. 4b.

One inherent advantage of RNA-seq over microarray-based analysis is the identification of splice variants and isoforms. Hence both RNA-seq samples were analyzed for expressed isoforms focusing on genes which are known to be important for the maintenance of the undifferentiated and pluripotent state of both hESCs and induced pluripotent stem cells (Table 1).

**Table 1:** Detected isoforms of selected human embryonic stem cell marker genes

Gene	Nearest ref ID	Locus	Length	Sample1 FPKM	Sample2 FPKM	Number of isoforms
DNMT3B				212.59	132.05	4/7 (13)
	NM_006892	chr20:31350190-31397167	789	9.71	4.87	
	NM_006892	chr20:31350190-31397167	721	0.00	3.00	
	NM_006892	chr20:31350190-31397167	3463	18.81	0.00	
	NM_006892	chr20:31350190-31397167	4131	0.00	21.60	
	NM_006892	chr20:31350190-31397167	4203	175.74	0.54	
	NM_006892	chr20:31350190-31397167	4336	8.33	65.00	
	NM_175848	chr20:31350190-31397167	4276	0.00	29.39	
	NM_175849	chr20:31350190-31397167	4087	0.00	7.65	
NANOG				3.91	6.62	1/1 (1)
	NM_024865	chr12:7941994-7948655	2089	3.91	6.62	
POU5F1				507.35	484.58	3/2 (21)
	NM_001173531	chr6:31132113-31138451	1247	156.01	0.00	
	NM_002701	chr6:31132113-31138451	1401	348.96	483.12	
	NM_203289.6	chr6:31132113-31138451	1733	2.38	1.46	

Gene expression is shown in FPKM values including detected isoforms. Number of detected isoforms for RNA-seq sample1 and sample2 are separated by dash; total number of known isoforms in brackets

### Ultra-low input whole genome sequencing

The sequencing of DNA was performed from the same sample as the sequencing of mRNA. The DNA contained in the human embryonic stem cells was collected during magnetic coupling of the mRNA/oligo-dT complexes and before cDNA synthesis was performed (Fig. 1a). The retained DNA was subjected to Phi29 mediated whole genome amplification (WGA) producing high molecular mass DNA. The WGA DNA was subjected to sonication (range 200–250 base pairs), followed by paired-end fragment library preparation and multiplex sequencing on the same flow cell as the mRNA samples on an Illumina HiSeq instrument. In total 190.5 million reads corresponding to the DNA of sample 1 were obtained. From these 153.5 million reads (80.6 %) could be mapped to hg19, giving rise to an average 6-fold genome coverage. The coverage for individual chromosomes ranged from 3-fold coverage for the X-chromosome to 13-fold coverage for chromosome 19 respectively. Furthermore chromosome coverage on a single base pair resolution was found to be ≥90.0 % for twelve chromosomes (chromosomes 2–5, 7, 10–12, 17–20). Lowest coverage was observed for the Y-chromosome with 34.2 % of sequenced bases. The average coverage for the full set of chromosomes was 82.8 % sequenced base pairs. A sequencing coverage overview over all chromosomes is presented in Fig. 4a.

## Discussion

To date the vast majorities of analyses of minute amounts of cell material down to single cells for next generation sequencing are still limited to either transcriptome or genome sequencing [9, 10]. Aiming the great advantages of a combined analysis, latest studies include different approaches for genome and transcriptome sequencing of the same cell [18, 19, 26]. Within the pre-NGS era the analysis of single cells was performed by a combination of microarray-based techniques for gene expression and comparative genomic hybridization to study genomic aberrations from the same cell [27]. Enabling a profound transcriptome as well as genome analysis by next generation sequencing from minute amounts of sample material will have many applications and will enable the study of rare cells. These rare cells may originate from cell-type specific differentiation of stem cells, but also from cancer tissue [22], circulating tumor cells [28] and early embryonic development [11] amongst others. Furthermore, it is known that micro-heterogeneity plays a fundamental role not only for stem cell function but for many biological processes [29]. Having tools to dissect these micro-heterogeneities on a transcriptomic as well as genomic level can help to further understand the underlying function of biological processes and might become of clinical relevance not only in cancer therapies but also in personalized medicine like pharmacogenomics or prenatal diagnosis.

Our results demonstrate that a combined transcriptome and genome analysis is feasible from minute amounts of sample material. Sequencing was performed from 150–200 hESC which equals about 200 pg total RNA according to the findings of Islam et al. [30] and about 1 ng of DNA for a diploid genome. In our approach the developed method for combined transcriptome and genome sequencing generated robust and quantitative data for studying gene expression, isoforms etc. on one hand and the genome on the other hand. This appears an especially advantageous situation in the case of studying cancer where remarkable chromosome instability causes genome heterogeneity and is directly linked to alterations in transcriptome dynamics [31]. The effect of genomic copy number variations and SNPs on the transcriptome has been investigated with an integrative approach [32]. A further elucidation of the impact of genomic alterations on the phenotype of a cell can be analyzed preferentially by the presented method, where DNA and RNA are sequenced from the very same cell material.

Obtaining full length coverage of expressed genes is vital for the identification of isoforms and splice variants. Therefore we specifically evaluated the average coverage along Refseq genes from the 5'- to the 3'-end. We found noteworthy variation in terms of average coverage from the 5'- to the 3'-end with significant more reads observed at the 3′-end of the transcript. Hence our RNA-seq data reflect a bias towards the 3'-end mainly attributed to the application of oligo-dT primed cDNA preparation. This finding is in agreement with findings of other groups who analyzed minute amounts of sample material down to single cells [1, 11, 28, 33]. Other protocols offer highly multiplexed single-cell sequencing, nevertheless only a limited number of bases either from the 5'-end [30] or the 3'-end [34] are sequenced to enable mainly gene expression studies. The duplication rate for mapped reads found in both RNA-seq samples is in line with comparable approaches eg by Adiconis et al. [35] where also an oligo-dT RNA preparation was performed and a duplication rate for low input samples of ~20 % (SMARTseq) and ~90 % (TRUseq) was reported. In their publication Dey et al. [18] do not present any information about duplicate reads observed in DNAseq and RNAseq, presumably due to their unique combined RNA and DNA amplification approach. The number of duplicate read counts observed by Macaulay et al. [19] ranged from 10 to <50 % for genome sequencing depending on cell line and cell number analyzed, unfortunately no read count for duplicate reads are given for RNA-seq, only the number of total and mapped reads are presented. Therefore the duplicate reads we found for RNA-seq seem currently more or less normal, nevertheless it is desirable to reduce the number in future, to make sequencing more efficient.

Purification of mRNA from the much more abundant ribosomal RNA using magnetic micro beads coupled to oligo-dT primers has become a widely established method in the last years. The recovery of complementary poly-T sequences is relatively high, whereas non-target RNAs represent an insignificant part of the enriched molecules [36]. Beyond the high target selectivity, the practicability and short assay duration of the method, further advantages are its compatibility to modifications. It has been shown that chemical conditions can also be changed to preserve proteins in their native state for further proteomic analyses [37]. However, the abovementioned 3′ skew bias has been faced in different approaches, like the cDNA transcription using SMARTer [28] or the additional mRNA enrichment by its 5′cap [38, 39]. The latter procedure also deals with the intricate capturing of mRNAs with short poly-A tails since its length might be influenced in the course of translational control [40]. Some new protocols avoid a physical separation of RNA from DNA because it might be detrimental when automatic liquid handling is conducted in small volumes. In such approaches, the oligo-dt primer contains an additional barcode sequence to identify the amplified cDNA within the pool of genomic DNA [41]. The limitation of all methods using oligo-dT primer to target polyadenylated mRNA is the inability to detect microRNAs or lncRNAs, which are also of importance for the transcriptome and the cell’s phenotype.

All current NGS platforms require prior amplification of DNA if minute amounts of DNA are analyzed. Novel approaches specifically utilized for single-cell whole genome amplification promise an improved genome coverage [17]. However, compared to established methods locus dropout is still observed and comparable to the results we obtained for whole genome sequencing. Alternatively a transposon-mediated library preparation strategy omitting any amplification reaction prior to NGS library preparation [8] may offer an alternative approach for the method presented here.

Several aspects of the presented method offer room for improvement. Beyond the separation of the mRNA the preparation of amplified cDNA as well as WGA DNA involves many discrete steps which are prone for technical variation, such as the magnetic coupling and binding of mRNA to the column, the elution of DNA from the column, the PCR amplification of the double-stranded cDNA and library preparation which adds another step of PCR amplification. Secondly, retaining full length coverage of expressed RNAs in ultra-low input preparations is especially challenging. Furthermore locus drop out on the genomic DNA is more likely to occur with decreasing cell numbers within the WGA reaction. All these technical challenges are exacerbating if the cell number is decreased down to single cells and all currently available methods need to cope with [10].

## Conclusion

In conclusion the presented approach for combined ultra-low mRNA and whole-genome sequencing from minute amounts of starting material offers new possibilities for many applications where limited material is available. Furthermore it enables one to directly study both the transcriptome and genome in one analytical approach from the same sample material which might be of interest for both basic as well as clinical research.

## Methods

### Cell culture and cell picking

Human embryonic stem cells (line H1) were obtained from WiCell Research Institute. Cells were cultured in 6-well-plates (TPP) coated with Matrigel (Becton Dickinson) on Mitomycin C-inactivated mouse embryonic fibroblasts (MEFs) as described before [42]. After 1 week, undifferentiated colonies were mechanically fragmented using the StemProEZPassage Disposable Stem Cell Passaging Tool (Invitrogen, cat# 23181–010) according to the recommendations of the manufacturer, leading to squares of relatively uniform size (ca. 200 μm × 200 μm, see Fig. 1b). Fragments from the middle of undifferentiated colonies were detached using a non-rotatable cell spatula (TPP, cat#99010) under microscopic control (Stereo microscope Leica MZ9.5 with cold light source KL 1500 LCD; Leica Microsystems) and sterile conditions inside a HERAguard® HPH 9 Laminar flow clean bench (Heraeus). Detached single squares were individually isolated by very gentle aspiration using a sterile 20 μl filter pipette tip (Biozym Scientific) and used for further downstream processing.

### Immunocytochemistry

Cells were fixed with 4 % paraformaldehyde (Electron Microscopy Sciences) in PBS (Gibco/Invitrogen) for 15 min, washed two times with PBS and then stained as described before [42]. Primary antibodies: anti-OCT3/4 (C-10) Mouse monoclonal antibody (Santa Cruz Biotechnology, cat#sc-5279) and anti-NANOG Goat polyclonal antibody (R&D Systems, cat#AF1997). Secondary antibodies: anti-Mouse IgG (H + L) (from chicken) labelled with red-fluorescent Alexa Fluor594 (Invitrogen, cat#A-21201) and anti-Goat IgG (H + L) (from donkey) labelled with green-fluorescent Alexa Fluor488 (Invitrogen, cat#A-11055). Nuclei were counterstained with DAPI. Fluorescence microscopy and photographing was performed using Axiovert 200 M (Zeiss) and Software AxioVision Rel. 4.8 (Zeiss).

### Ultra low input cDNA and WGA-DNA preparation

Preparation and amplification of nucleic acids were performed with a customized version of the μMACS SuperAmp Kit (Miltenyi Biotec); if not mentioned explicitly, procedures were according to the manufacturer protocol. The protocol is based on magnetic coupling of mRNA and retaining the nucleic acid in low volume flow-through columns for greatly simplified handling. For selective mRNA isolation, magnetic micro beads coupled to oligo-dT primers were applied. To retain the genomic DNA the eluates from the first two washing steps after loading the cell lysate onto the column were collected into a 1.5 mL reaction tube for later DNA precipitation and whole genome amplification. On column cDNA synthesis was performed at 42 °C for 60 min according to the following protocol by applying the total reaction master mix onto the column: 20 μL contained 2 μL 10× Reverse Transcriptase Buffer (Ambion), 0.5 mM dNTPs, 1 μg T4 Gene 32 Protein (NEB), 400 U M-MLV Reverse Transcriptase (Enzymatics), 20 U RNase Inhibitor (Ambion). After collection of magnetic beads containing synthesized cDNA by centrifugation and 3'-tailing according to the manufacturer, PCR amplification was performed. To the 3'-tailing reaction, in total 30 μL, the following PCR master mix was added: 76.5 μL PCR master mix contained 14 μL 5× Phusion HF buffer (Finnzymes), 0.5 mM dNTPs, 60 μL resuspended μMACS SuperAmp PCR mix, 2 U PhusionTaq (Finnzymes); the following cycling conditions were applied on a PTC-200 (MJ Research) thermal cycler: 78 °C for 30 s, 95 °C for 1 min, [98 °C for 3 s, 64 °C for 30 s, 72 °C for 2 min]× 40 cycles, 72 °C for 5 min.

Amplification of genomic DNA was performed with the REPLI-g Midi Kit (Qiagen) for 16 h at 30 ° C according to the manufacturer’s recommendations. Before whole genome amplification, DNA was ethanol precipitated by adding 0.1 volumes of 3 M sodium acetate solution and 5 μg glycogen (Ambion) to 1 volume of DNA sample. After precipitation the pellet was resuspended in 10 μL of Elution Buffer (Qiagen).

### Library preparation and NGS

Library preparation for next generation sequencing was performed according to the Illumina TruSeq DNA Sample Preparation Guide with the Low-Throughput protocol. The indexed paired‐end libraries had an insert size in the range of 150–300 base pairs. Subsequent pooling of the samples with a ratio of 3:1:1 (wgaDNA:mRNA1:mRNA2) cluster generation and DNA sequencing was performed on a single lane of an IlluminaHiSeq instrument with a 100 base pair paired-end sequencing chemistry.

### Mapping and data analysis

#### RNA-seq mapping

RNA-seq data was mapped to the human genome by Tophat v1.3.3. Prebuild bowtie index files and annotations in GTF-format were downloaded from Illumina’s iGenomes ftp-server (ussd-ftp.illumina.com/Homo_sapiens/UCSC/hg19/). Duplicate read counts were estimated on mapped reads using Picard (http://broadinstitute.github.io/picard/). To compare Tophat mappings to Illumina BeadArray data, we considered only reads that mapped to annotated exons (UCSC genes) and reached peak coverage of 5 or higher. Furthermore we restricted the analysis to Refseq genes.

#### Whole genome mapping

Genomic DNA sequencing reads were mapped using bowtie with the same parameters that Tophat uses for its first mapping round (−v 2). Duplicate read counts were estimated on mapped reads using Picard (http://broadinstitute.github.io/picard/). Genomic coverage was visualized as Manhattan plot via the *mhtplot* function from the R package *gap*.

#### Transcriptome read coverage analysis

Coverage was calculated via the IGVtools command count [43] from the exon aligned BAM files for RNA sample1 and sample2 using default settings. For transcript coverage window size 25 was used, for genomic coverage window size 10,000 was used. Transcript calculations were based on exon unions of human genes from ENSEMBL V74 for plus and minus strand separately. Each transcript was divided into 40 equally sized bins according to the method of [28]. To compensate for missing values data points corresponding to the 40 bins were determined by interpolation of the IGVtools results via cubic spline curve fitting (function *spline*) from the statistical software package R. Resulting values were normalized via division by the maximum. These transcript coverages were averaged for transcript size intervals 0 kb −1 kb, 1 kb −2 kb, 2 kb −3 kb, 3 kb −4 kb, 4 kb −5 kb and 5 kb −15 kb. Plus and minus strand were summarized by calculating mean values for all transcript size intervals. Finally, mean values and standard deviations were determined between the two RNA samples shown in the coverage plots. Boxplots of sequenced mRNA length were plotted by the R package.

### Overlap between microarray and sequencing measurements

Congruence of Illumina microarray and next generation sequencing experiments was determined in terms of overlapping genes and overlapping categories found via Consensus Pathway Data Base (CPDB) overrepresentation analysis [44]. Genes were considered significantly expressed when the FPKM (Fragments per kilobase of exon per million fragments mapped) values were greater than 0.5 in the sequencing experiments or Illumina detection *p*-value was less than 0.05. Additionally, these genes were subjected to a CPDB overrepresentation analysis using pathways from KEGG, Reactome, BioCarta and Wikipathways and the resulting categories were compared using a threshold of 0.05 for *p*-values adjusted via the Benjamini-Hochberg method. The results were displayed in Venn diagrams from R package Vennerable.

### Cluster analysis of pluripotency associated genes

Illumina average signals from microarray experiment and FPKM values from sequencing experiments of sample1 and sample2 were compared with respect to pluripotency associated genes [29] which were expressed in the microarray experiment (detection *p*-value < 0.05) and in the sequencing experiments (FPKM > 0.5). Logarithmic values (base 2) of these measurements were quantile normalized and subjected to cluster analysis via R heatmap2 function using Euclidean distance as distance measure.

## Availability of supporting data

The data sets supporting the results of this article are available in the GEO repository at GEO accession number GSE69471 (http://www.ncbi.nlm.nih.gov/geo/query/acc.cgi?acc=GSE69471).
